# Cerebellum Involvement in Dystonia During Associative Motor Learning: Insights From a Data-Driven Spiking Network Model

**DOI:** 10.3389/fnsys.2022.919761

**Published:** 2022-06-16

**Authors:** Alice Geminiani, Aurimas Mockevičius, Egidio D’Angelo, Claudia Casellato

**Affiliations:** ^1^Department of Brain and Behavioral Sciences, University of Pavia, Pavia, Italy; ^2^Brain Connectivity Center, IRCCS Mondino Foundation, Pavia, Italy

**Keywords:** cerebellum, dystonia, learning, motor dysfunction, modeling, spiking neural networks, simulations

## Abstract

Dystonia is a movement disorder characterized by sustained or intermittent muscle contractions causing abnormal, often repetitive movements, postures, or both. Although dystonia is traditionally associated with basal ganglia dysfunction, recent evidence has been pointing to a role of the cerebellum, a brain area involved in motor control and learning. Cerebellar abnormalities have been correlated with dystonia but their potential causative role remains elusive. Here, we simulated the cerebellar input-output relationship with high-resolution computational modeling. We used a data-driven cerebellar Spiking Neural Network and simulated a cerebellum-driven associative learning task, Eye-Blink Classical Conditioning (EBCC), which is characteristically altered in relation to cerebellar lesions in several pathologies. In control simulations, input stimuli entrained characteristic network dynamics and induced synaptic plasticity along task repetitions, causing a progressive spike suppression in Purkinje cells with consequent facilitation of deep cerebellar nuclei cells. These neuronal processes caused a progressive acquisition of eyelid Conditioned Responses (CRs). Then, we modified structural or functional local neural features in the network reproducing alterations reported in dystonic mice. Either reduced olivocerebellar input or aberrant Purkinje cell burst-firing resulted in abnormal learning curves imitating the dysfunctional EBCC motor responses (in terms of CR amount and timing) of dystonic mice. These behavioral deficits might be due to altered temporal processing of sensorimotor information and uncoordinated control of muscle contractions. Conversely, an imbalance of excitatory and inhibitory synaptic densities on Purkinje cells did not reflect into significant EBCC deficit. The present work suggests that only certain types of alterations, including reduced olivocerebellar input and aberrant PC burst-firing, are compatible with the EBCC changes observed in dystonia, indicating that some cerebellar lesions can have a causative role in the pathogenesis of symptoms.

## Introduction

In the sensorimotor system, the cerebellum plays a key role in motor learning and control (Ito, 2000). Thanks to synaptic plasticity at multiple connection sites (Hansel et al., 2001), the cerebellar output adapts and fine-tunes the amplitude and timing of motor responses in changing and perturbed environments (Marr, 1969; Albus, 1971). A typical cerebellum-driven protocol is Eye-Blink Classical Conditioning (EBCC), where a neutral stimulus, e.g., a sound, is provided (Conditioned Stimulus – CS), followed by a time-locked aversive stimulus, e.g., an air puff on the eye (Unconditioned Stimulus – US), causing a motor response – eye closure. After repeated paired presentations of these stimuli, the cerebellum drives the anticipation of the motor response before the US onset (Conditioned Response – CR; Freeman and Steinmetz, 2011). EBCC is widely used to investigate the neural correlates of cerebellum-driven behavior and also as a clinical biomarker of diseases involving the cerebellar circuit (Steinmetz et al., 2001). For example, EBCC is impaired in spinocerebellar ataxia, but also in pathologies not thought to involve cerebellar regions. Patients with Alzheimer’s Disease or neurodevelopmental disorders show impaired EBCC learning, suggesting instead that cerebellar alterations may play a role in these pathologies (Woodruff-Pak et al., 1996; Reeb-Sutherland and Fox, 2015). For other brain diseases, the involvement of the cerebellum is still debated. This is the case of dystonia, a neurological motor disorder characterized by sustained or intermittent muscle contractions that generate repetitive movements, abnormal fixed postures, or both (Albanese et al., 2013). Dystonic movements are typically patterned, twisting, or tremulous, and are often initiated or worsened by voluntary action. It is estimated that dystonia is the third most common movement disorder after Parkinson’s disease and essential tremor (Defazio, 2010), and it can have a significant negative impact on the general functioning and quality of life (Camfield et al., 2002). Understanding the pathological neural mechanisms that underlie dystonia is essential to design precise treatment strategies.

### Dystonia and the Cerebellum

Although typically associated with dysfunctional basal ganglia (Tarsy and Simon, 2009), dystonia is now commonly regarded as a network disorder (Neychev et al., 2011), involving multiple brain areas and their connections. One of them is the cerebellum (Shakkottai et al., 2016), which is a fundamental part of the motor control system (Marr, 1969; Albus, 1971). In humans, imaging studies have shown cerebellar abnormalities in dystonic patients, and impairments in EBCC learning curves have been reported (Teo et al., 2009; Bologna and Berardelli, 2017). EBCC alterations in dystonia are debated and may not be discriminant of dystonia itself, but might be specific to some dystonia types (Sadnicka et al., 2022).

In rodents, pharmacological and genetic manipulations altering the cerebellum can result in dystonic movements (Isaksen et al., 2017). Damages to signal transmission from the inferior olive to Purkinje cells cause severe motor impairment, and multiple features of dystonia were observed in mouse models where olivocerebellar transmission was silenced (White and Sillitoe, 2017). In other rodent models of dystonia, aberrant firing patterns or synaptic alterations of Purkinje cells have been reported (Fremont et al., 2015; Vanni et al., 2015).

At present, a multi-regional motor network model for dystonia has been proposed stating that dystonia can arise from disruptions in multiple different brain regions, including the basal ganglia, the cerebellum and the motor cortex along with their connections (Prudente et al., 2014; Schirinzi et al., 2018). Cerebellar alterations may play a distinctive role in the generation of dystonic movements or could induce aberrant activity in the basal ganglia through subcortical connections. Otherwise, cerebellar changes in dystonia may represent a compensation to dysfunctions in other areas. Therefore, the exact role of cerebellar alterations still remains to be elucidated.

### Computational Models of the Cerebellum

Computational models embedding realistic neuronal network properties and reproducing motor functions provide a new tool to investigate the neural mechanisms of behaviors in physiological and pathological conditions. Plastic cerebellar spiking models embedded in closed-loop control systems can simulate cerebellum-driven sensorimotor tasks by using the underlying neurophysiological mechanisms (Yamazaki and Tanaka, 2007; Antonietti et al., 2016; Geminiani et al., 2019b). Specific lesions can be applied to these data-driven Spiking Neural Networks (SNNs) to investigate the causal relationships between neural alterations and the disease symptoms. For example, simulations predicted that alterations of synaptic plasticity in the cerebellar cortex could impair EBCC in different forms of cerebellar ataxia and that synaptic plasticity in the deep cerebellar nuclei was a possible compensatory mechanism (Geminiani et al., 2018a). Moreover, in autism spectrum disorder, simulations suggested that a higher CR learning rate could reflect a reduced number of Purkinje cells (Trimarco et al., 2021).

Here, we applied this approach to investigate the involvement of the cerebellum in dystonia through simulations of EBCC driven by a bioinspired SNN model of the cerebellum. The aim is to understand how specific cerebellar alterations impact EBCC in controlling stimuli association and timing (Teo et al., 2009). Three types of alterations observed in rodent models of dystonia were modeled: (i) reduced olivocerebellar connectivity (Ledoux and Lorden, 2002; White and Sillitoe, 2017), (ii) aberrant Purkinje cell firing (burst-firing; Ledoux and Lorden, 2002; Hisatsune et al., 2013), (iii) imbalanced synapse density on Purkinje cells (Vanni et al., 2015).

## Materials and Methods

### Olivocerebellar Spiking Neural Network

The cerebellar model was a Spiking Neural Network (SNN) including the cerebellar cortex, the deep cerebellar nuclei (DCN) and the inferior olive (IO), forming a cerebellar microcomplex. It reproduces a microcomplex with Zebrine-negative Purkinje cells (characterized by a higher intrinsic excitability), which is considered the primary mechanism responsible for EBCC learning (Wu et al., 2019).

**FIGURE 1:**
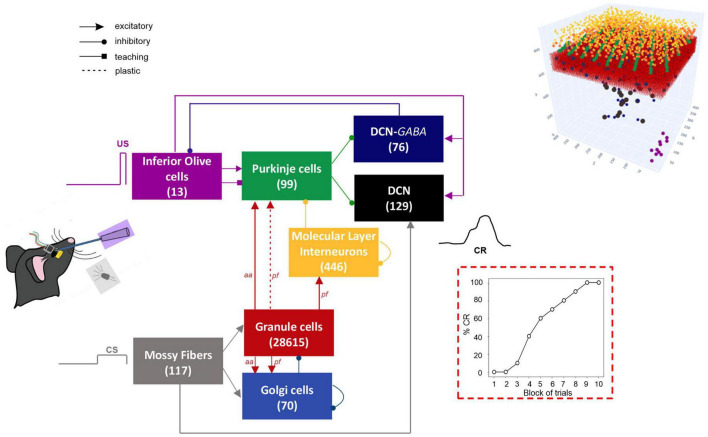
Block diagram of the olivocerebellar SNN and the EBCC signals (CS; US; CR). On the right, the 3D network volume with the placed neurons is depicted (the Golgi cells are immersed in the granular layer). The number of cells is reported in each population box.

The network architecture was built through a data-driven reconstruction process using the Brain Scaffold Builder (BSB; RRID:SCR_008394, version 3.8+). Neurons were placed in the defined volume and connected based on spatial information (cell densities, oriented morphologies and synapse densities; de Schepper et al., 2021). In the resulting SNN, at the input stage, mossy fibers (*mfs*) excite Granule Cells (GrCs) and Golgi cells (GoCs); GrCs provide excitatory input to GoCs and to Purkinje cells (PCs) through ascending axons (*aas*) and parallel fibers (*pfs*), and excitatory input to Molecular Layer Interneurons (MLIs) through *pfs.* MLIs in turn inhibit PCs. DCN contains two neural populations, (i) GAD-negative large cells (DCN), which are large glutamatergic neurons projecting outside the cerebellum, and (ii) DCN-GABA, which are GABAergic neurons forming the olivo-cerebellar loop. The output of the cerebellum, DCN neurons, receives direct excitation from *mfs* and inhibition from PCs. IO neurons trigger burst-pause responses in PCs through excitatory climbing fibers (*cfs*) which also excite DCN and DCN-GABA (Figure 1). Neurons were modeled as Extended-Generalized Leaky Integrate and Fire point neurons (E-GLIF) with parameters optimized as in Geminiani et al. (2018b,2019a). Neural connections were modeled as conductance-based synapses, with delays extracted from literature and weights tuned to reproduce physiological firing rates in mice at rest (Geminiani et al., 2019b). Connections between *pfs* and PCs were plastic, according to an *ad hoc* Spike Timing Dependent Plasticity rule, driven by the IO teaching signal (Casellato et al., 2014; Luque et al., 2016): concurrent spikes at *pfs* and IOs caused Long-Term Depression (LTD) at the corresponding *pf*-PC synapses, while *pfs* spikes alone caused Long-Term Potentiation (LTP), consistently with experimental observations (Sakurai, 1987; Coesmans et al., 2004).

Simulations were run in NEST (version 2.18), a software simulator for spiking neural networks (Gewaltig and Diesmann, 2007; Eppler et al., 2009; Jordan et al., 2019; RRID:SCR_002963). All simulations were carried out on a PC provided with Intel^®^ Core™ i7-8750H CPU @ 2.20GHz with 16.0 GB RAM in the Operating System Ubuntu 20.04.2 LTS.

### EyeBlink Classical Conditioning Protocol

To simulate EBCC, the cerebellar SNN was embedded in a closed-loop control system, with learning intrinsically driven by synaptic plasticity (Antonietti et al., 2016). The sensory and motor signals were encoded in the corresponding cerebellar neural populations according to experimental evidence. *Mfs* conveyed the CS, which was simulated as a non-recurrent 40-Hz spike train delivered to each individual *mf*, lasting 280 ms. The pattern was chosen to generate GrC low-frequency sparse coding which is supposed to be fundamental for cerebellar learning (Schweighofer et al., 2001). The US was delivered to IOs as a 500-Hz burst lasting 30 ms and co-terminating with the CS.

The eyelid closure signal was decoded from DCN spikes (see paragraph below *Data Analysis*).

The protocol included 100 trials (10 blocks of 10 trials each) in a row, with 280 ms of stimulation and a 720-ms pause of baseline activity (total trial duration of 1000 ms).

### Simulations of Cerebellar Alterations in Dystonia

Three specific localized lesions were applied to the control model.

**(i) Reduced olivocerebellar input**. Studies in mutant dystonic (dt) rats suggest that reduced IO signaling could be related to dystonia (Stratton and Lorden, 1991; Ledoux and Lorden, 2002). Specifically, decreased PC complex spike firing was observed from single cell recordings (Ledoux and Lorden, 2002), which would result from reduced IO activity. In a more recent study, White and Sillitoe devised a mouse model with genetic silencing of IO, causing the elimination of PC complex spikes as well as severe dystonia (White and Sillitoe, 2017). Complete silencing of IO input would be expected to abolish EBCC acquisition (Zbarska et al., 2007), as it is crucial for the US circuitry and for *pf*-PC plasticity. This type of alteration was modeled in our cerebellar SNN by re-tuning two factors at the same time, (a) by disconnecting the IO teaching signal from a subset of PCs, (b) by reducing the strength in all the synapses between IOs and PCs. Three levels of damage were simulated: 25, 50, and 75% reduction on both factors.

**(ii) Aberrant burst-firing pattern of PCs**. It has been observed in a number of dystonic rodent models (Ledoux and Lorden, 2002; Hisatsune et al., 2013; Fremont et al., 2015; Washburn et al., 2019). Specifically, PC simple spike burst-firing was reported in dt rats (Ledoux and Lorden, 2002) and PC burst-firing with excessive and repetitive complex spike firing was observed in cerebellum-specific IP3R1 knock-out dystonic mice, which also exhibited increased IO activity (Hisatsune et al., 2013). Both types of burst-firing alterations were modeled in our cerebellar SNN. In one case (intrinsic burst-firing), PC burst-firing was obtained by directly stimulating PCs with intermittent 20-ms spike trains with random 20 or 30 ms pauses, as in experiments, and by reducing PC intrinsic firing. In the other case (IO-induced burst-firing), intermittent stimulation was provided to all IO neurons using 40-ms spike trains with 40 ms pauses, causing increased IO activity and inducing PC bursting as reported in IP3R1 knock-out mouse model of dystonia.

**(iii) Imbalanced PC synaptic densities** (from MLIs, *pf*s and IOs). It was reported in a mouse model of early-onset isolated dystonia, DYT1 (Vanni et al., 2015). This type of alteration was modeled in our cerebellar SNN by reducing the synapse density between *pf*-PC and MLI-PC, and increasing the synapse density between IO-PC, with proportions as in the experimental study. Two levels of impairment were explored (mild and severe): (a) %14 decrease in *pf*-PC, %39 decrease in MLI-PC,%32 increase in IO-PC, (b) %25 decrease in pf-PC, %71 decrease in MLI-PC, %57 increase in IO-PC. When computing the ratio between the number of excitatory and inhibitory synapses onto PCs (structural E/I balance), these mild and severe alterations corresponded to an increase from 62.3 in the control condition to 88.6 and 110.9, respectively.

### Data Analysis

The activity of PCs and DCN was monitored along task repetitions, as these two neuronal populations underwent firing changes driven by *pf*-PC plasticity. To assess intrinsic firing properties (firing rate and irregularity, respectively), we computed the number of spikes and the Coefficient of Variation of the Inter-Spike Intervals (CV_ISI_) in the baseline time window of the first trial, i.e., from the end of CS to the end of trial (from 280 to 1000 ms). To assess firing modulation, we computed the Spike Density Function (SDF) as the convolution of each cell’s spikes in every trial, with a Gaussian kernel of 20 and 10 ms for PCs and DCN, respectively (Dayan and Abbott, 2001). SDFs of the two populations were then computed by averaging SDFs of individual cells. We evaluated the population SDF in the CR time window, i.e., between 100 ms from the CS onset to the co-termination of CS and US (i.e., 280 ms from CS onset). Furthermore, to quantify learning in terms of neural activity, an index of *SDF change* was computed as the time-integral of SDF in the CR time window of the last block (averaged across the last 10 trials) subtracting the average activity in the first 100 ms after the CS onset. This quantifies the time-locked rate change, i.e., the modulation in the inter-stimuli-interval within each trial.

The motor output response (eyelid closure) was derived by applying a moving average filter with a 100-ms time window to SDF of the DCN population, obtaining a *filtered SDF*. A CR was detected when this motor output signal reached a fixed threshold of 4 Hz in the CR time window and remained above the threshold. The CR time window did not include the first 100 ms from the CS onset because non-EBCC related responses are supposed to occur there, according to experimental evidence (ten Brinke et al., 2015). The CR threshold was set at 4 Hz to have 70% of CRs in the 6th block of learning, as in EBCC experiments on control mice (de Oude et al., 2021).

We computed %CR in each block of 10 trials, and the timing of the CR onset (advance with respect to the US onset). The CR onset was defined as the time instant when the motor output began monotonically rising.

For SDF change, baseline firing rate and CV_ISI_ distributions, outliers were excluded as the values falling outside the interval between Q1 – 1.5 * IQR and Q3 + 1.5 * IQR, where Q1 is the 1st quartile, Q3 is the 3rd quartile, IQR is the Inter-Quartile Range.

The Wilcoxon statistical test was applied to compare CR onset, SDF change, baseline firing rate and CV_ISI_ values between simulations of control and pathological conditions, with statistical significance at *p* < 0.01. The McNemar’s test with *p* < 0.01 was applied to %CR.

## Results

### Simulation of Physiological Conditions

**FIGURE 2:**
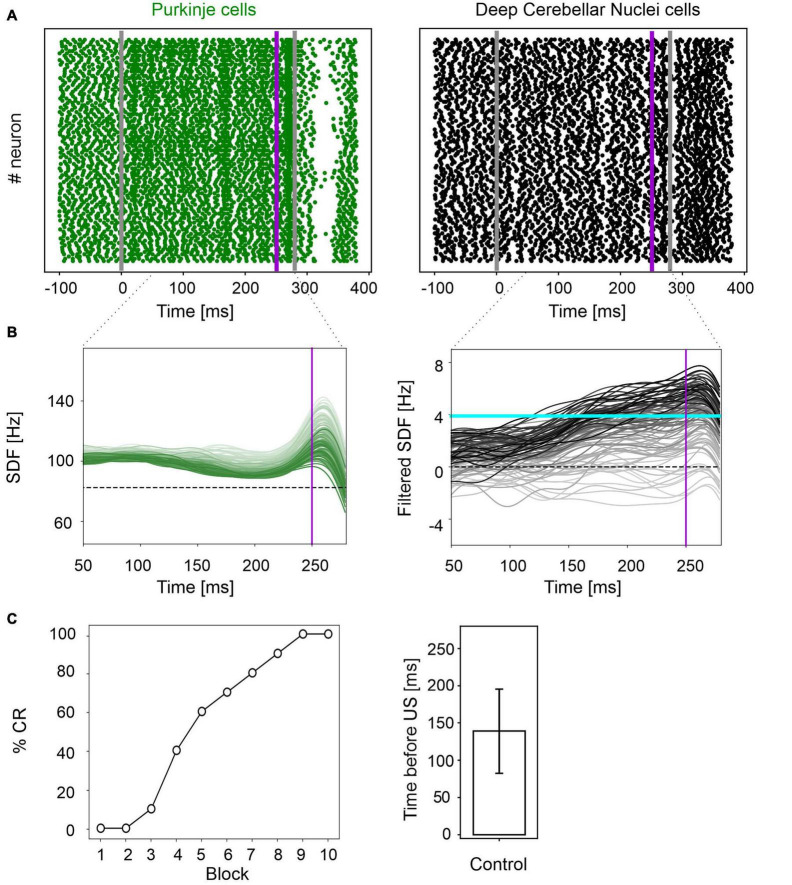
EBCC simulations in the control condition. **(A)** Exemplificative raster plots showing spiking activity of each cell (y axis) within PC (green) and DCN (black) populations in the first trial. Gray vertical lines represent the CS onset and end, purple line marks the US onset (which co-terminates with CS). **(B)** SDF in PC population and DCN motor output (averaging across cell SDFs) for each trial (0 ms is aligned with the CS onset). Increasing darkness of the lines corresponds to subsequent trials (the lightest is the first trial, the darkest is the last trial). Dashed horizontal line indicates baseline activity, solid horizontal cyan line (right panels) represents the CR threshold. Purple vertical line corresponds to US onset time. **(C)** Behavioral EBCC outcome: %CR along trial blocks (left panel), and CR timing with respect to US onset (right panel). Error bar indicates the standard deviation.

In physiological conditions, the model was able to reproduce the EBCC learning curve obtained in mice experiments (de Oude et al., 2021). In the first trial, PC spike pattern at the CS onset increased from basal discharge and remained constant during the entire inter-stimuli interval; then, during the US, a burst was generated, followed by a pause. PC pause released DCN neurons, which fired strongly, generating a blink after the US (Figure 2A and Supplementary Figure 1). Thanks to *pf*-PC LTD in the time window before the US, PC firing rate decreased throughout trials due to a progressive accentuation of the spike suppression (Figure 2B left, and Supplementary Figure 1). This caused a gradual release of DCN firing (i.e., DCN facilitation; Figure 2B right, and Supplementary Figure 1). Along the learning process, PC suppression became more and more evident. In the last trial, most of PCs (39 out of 99) underwent a significant suppression in the second half of the CR time window, while a few PCs (14 out of 99), due to LTP and intrinsic neuronal mechanisms, moved to an upstate at the beginning of the CR time window (Supplementary Figure 2).

Consequently, along the learning process, DCN facilitation yielded a progressive increase of the cerebellar motor output, triggering eye closures before the US onset (Figure 2C left). The resulting %CR curve matched the values obtained in experimental studies, reaching 70% in the 6th block of learning, and 100% in the 9th block. Also, the response timing was in the physiological range, with a CR onset of 139 ± 57 ms before the US (Figure 2C right).

### Simulation of Reduced Olivocerebellar Input

A reduced IO input to PCs caused insufficient modulation of PC and DCN activity, as well as a slower and reduced %CR learning curve. By further increasing the amount of damage, the behavioral dysfunction became more severe.

**FIGURE 3:**
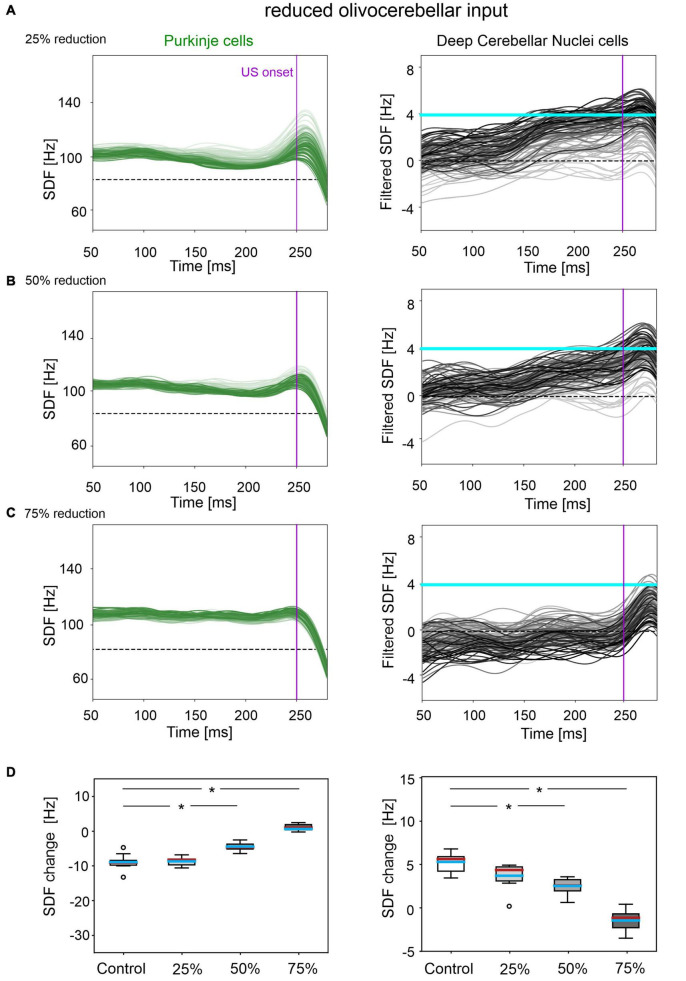
Neural activity underlying EBCC in the case of reduced olivocerebellar input by 25% **(A)**, 50% **(B)**, 75% **(C)**. SDF in PC population and DCN motor output (averaging across cell SDFs) for each trial (0 ms is aligned with the CS onset). Increasing darkness of the lines corresponds to subsequent trials (the lightest is the first trial, the darkest is the last trial). Dashed horizontal line indicates baseline activity, solid horizontal cyan line (right panels) represents CR threshold. Purple vertical line corresponds to US onset time. **(D)** SDF change in PC (left) and DCN (right) populations from the 10 trials in the last block, with respect to SDF value in the first 100 ms from CS onset. Cyan horizontal line indicates the mean, red horizontal line the median, box edges the 1st and 3rd quartile, whiskers the minimum and maximum accepted values, excluding outliers (indicated as circles). * corresponds to *p* < 0.01 in the Wilcoxon test.

**FIGURE 4:**
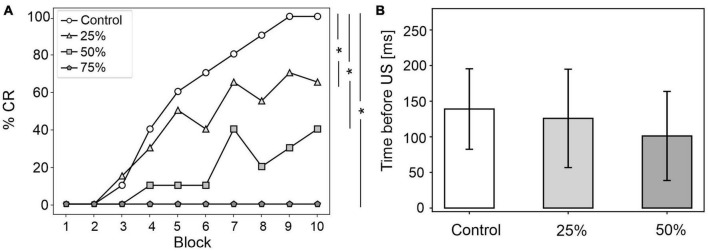
EBCC behavior in the case of reduced olivocerebellar input (25%, 50 and 75%), compared to the control condition. **(A)** %CR along trial blocks. **(B)** CR timing with respect to US onset. Error bars indicate the standard deviation. Impaired condition with 75% IO-PC reduction is not represented in B) as no CRs were acquired during the simulation. * corresponds to *p* < 0.01 in the McNemar’s test for %CR and the Wilcoxon test for the CR onset timing.

In all three tested damage levels, baseline properties of PCs and DCN were not altered, indicating a normal intrinsic neuron firing: firing rate and irregularity were not significantly different with respect to the control simulations (Supplementary Figure 3). With 25% decrease in IO-PC signaling, PC spike suppression was slightly less pronounced, resulting in a reduced DCN facilitation (Figure 3A). Overall, the SDF changes were comparable to control simulations (Figure 3D). Consequently, the model was still able to produce CRs, even if the acquisition was slower and more unstable than in the control network, with the maximum %CR reaching 70% (Figure 4A). As expected, compromising half of the IO-PC signaling amplified the effects (Figure 3B): PC and DCN SDF changes were significantly smaller than in control simulations (Figure 3D) and the motor output rarely reached the CR threshold. Consequently, CR acquisition was compromised, reaching a maximum of 40% (Figure 4A). With the highest level of damage, the activity of PC and DCN populations remained constant throughout the whole EBCC training protocol (Figure 3C). PC SDF change, on average, was close to 0, while mean DCN SDF change was even negative (Figure 3D). The motor output never reached the CR threshold before US onset, thus, no CRs were produced throughout the entire task (Figure 4A).

CR curves were significantly lower than in controls in all three levels of damage (total number of CRs in control = 56; in 25% damage = 30; in 50% damage = 17; in 75% damage = 0; McNemar’s *p* < 0.01 in all damage cases vs. control). Regression analysis proved a significant linear relationship between the damage level and the number of CRs (*R*^2^ = 0.98 and *p* = 0.01). CR onset was delayed but the difference did not reach statistical significance (in control = 139 ± 57 ms; in 25% damage = 126 ± 69 ms; in 50% damage = 101 ± 62 ms; in 75% damage: N/A. *p* > 0.01 in all damage cases vs. control; Figure 4B).

### Simulation of Aberrant Burst-Firing Pattern of PCs

The applied modifications to the cerebellar SNN transformed PC simple spike activity into burst-firing (Supplementary Figure 4). In the simulations with intrinsic PC burst-firing, PC activity was characterized by short intervals of increased spiking activity, separated by pauses. This led to a significant increase in mean PC CV_ISI_ when compared to the control condition (Supplementary Figure 5). Moreover, due to the altered PC firing, DCN also exhibited a corresponding burst-firing pattern and increased irregularity (Supplementary Figure 5). Baseline firing rates were statistically different from the control condition, but still in the physiological ranges for the activity of PCs and DCN of EBCC-related cerebellar modules (de Zeeuw and Ten Brinke, 2015; Beekhof et al., 2021).

**FIGURE 5:**
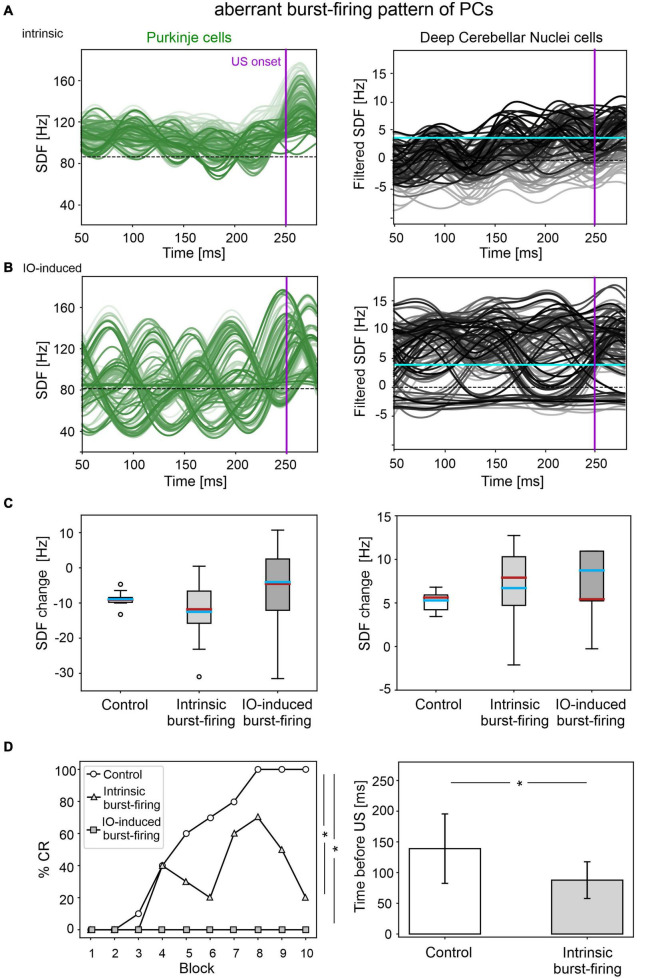
EBCC simulations in the case of aberrant burst-firing pattern of PCs. SDF in PC population and DCN motor output (averaging across cell SDFs) for each trial (0 ms is aligned with the CS onset), with intrinsic PC burst-firing **(A)** and IO-induced PC burst-firing **(B)**. Increasing darkness of the lines corresponds subsequent trials (the lightest is the first trial, the darkest is the last trial). Dashed horizontal line indicates baseline activity, solid horizontal cyan line (right panels) represents CR threshold. Purple vertical line corresponds to US onset time. **(C)** SDF change in PC (left) and DCN (right) populations from the 10 trials in the last block, computed in the CR window with respect to SDF value in the first 100 ms from CS onset; * corresponds to *p* < 0.01 in the Wilcoxon test. **(D)** Behavioral EBCC outcome, i.e., %CR along trial blocks (left panel), and CR timing with respect to US onset (right panel), compared to the control condition. Error bars indicate the standard deviation. CR onset is not represented for the IO-induced PC burst-firing case, as no CRs were acquired during the simulation. * corresponds to *p* < 0.01 in the McNemar’s test for %CR and the Wilcoxon test for the CR onset timing.

The population firing of PCs and the DCN motor output showed irregular oscillations (Figure 5A). PCs and DCN were more variable in the impaired than in the control model, but the difference was not statistically significant (Figure 5C). The motor output in some trials exceeded threshold already in the first 100 ms, i.e., before the plausible time window for CRs. This improperly timed response was not considered as CR. Consequently, CR acquisition was impaired (Figure 5D), as the proportion of trials with detected CR was significantly different between control and impaired conditions (control: 56; impaired: 26, McNemar’s *p* < 0.01). The maximum %CR was 60% in the 8th block. When generated, the CRs were significantly delayed (control: 139 ± 57 ms, impaired: 113 ± 33 ms, before US onset; *p* < 0.01).

In the simulations with IO-induced PC burst-firing, excessive olivocerebellar signaling resulted in PC burst-firing patterns: sharp increases in PC firing induced by IO input were present, which were followed by pauses (Supplementary Figure 4). This PC burst-firing contributed to abnormal burst-firing patterns of DCN, which showed pauses during increased PC activity as well as increased firing during PC pauses. Thus, the irregularity of PC and DCN neurons was significantly higher than in the healthy network; PC baseline activity was similar and DCN baseline was slightly different from the control condition (Supplementary Figure 5), but still in the physiological ranges reported for the activity of DCN in EBCC-related cerebellar modules (de Zeeuw and Ten Brinke, 2015; Beekhof et al., 2021).

PC and DCN firing patterns during trials were highly disorganized in the burst-firing intervals, with DCN also exhibiting increased firing rates with improper modulation (Figure 5B). PC and DCN SDF changes were more variable in the impaired model when compared to the control one, but not resulting in significant differences (Figure 5C). No CR acquisition was observed in this condition (Figure 5D).

### Simulation of Imbalanced PC Synaptic Densities

**FIGURE 6:**
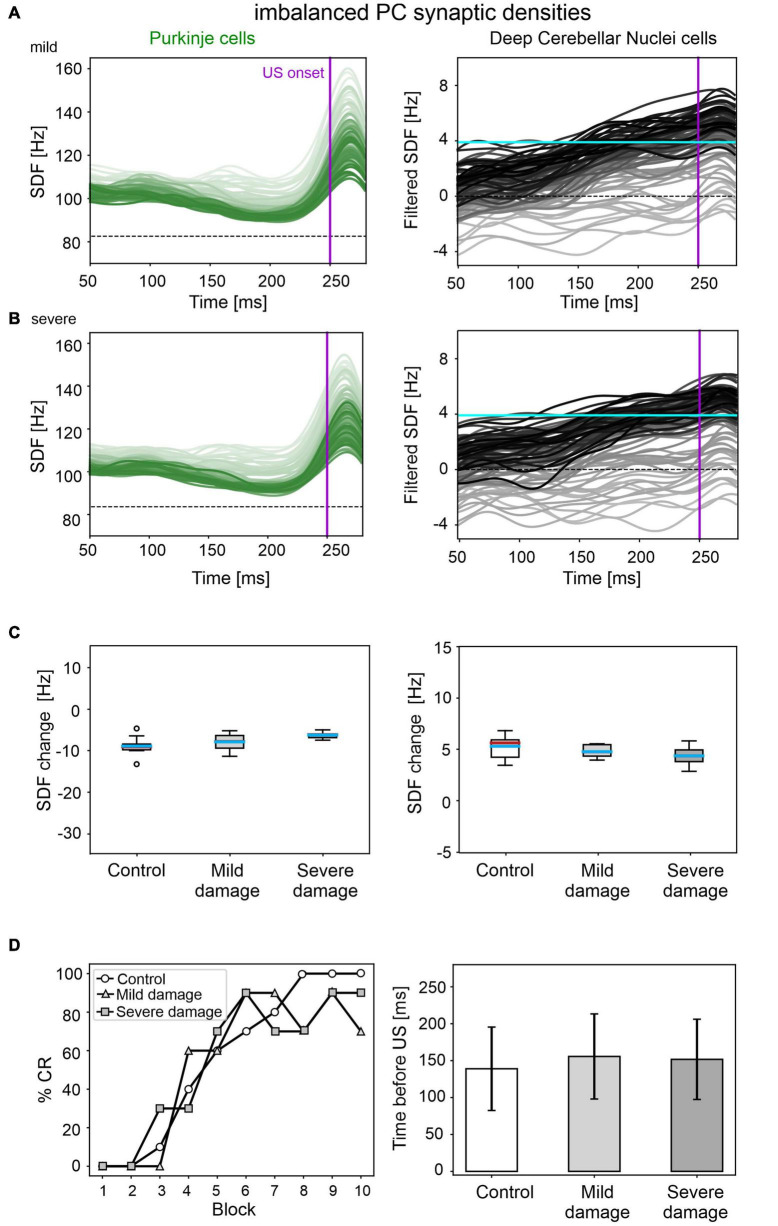
EBCC simulations in the case of imbalanced PC synaptic densities. SDF in PC population and DCN motor output (averaging across cell SDFs) for each trial (0 ms is aligned with the CS onset), with mild **(A)** and severe damage **(B)**. Increasing darkness of the lines corresponds to subsequent trials (the lightest is the first trial, the darkest is the last trial). Dashed horizontal line indicates baseline activity, solid horizontal cyan line (right panels) represents CR threshold. Purple vertical line corresponds to US onset time. **(C)** SDF change in PC (left) and DCN (right) populations from the 10 trials in the last block, with respect to SDF value in the first 100 ms from CS onset. Cyan horizontal line indicates the mean, red horizontal line the median, box edges the 1st and 3rd quartile, whiskers the minimum and maximum accepted values, excluding outliers (indicated as circles). **(D)** Behavioral EBCC outcome, i.e., %CR along trial blocks (left panel), and CR timing with respect to US onset (right panel), compared to the control condition. Error bars indicate the standard deviation.

A mild damage on PC synaptic densities was implemented with 14% reduction in *pf*-PC, 39% reduction in MLI-PC and 32% increase in IO-PC. The model produced comparable results as in the control condition (Figure 6). PC suppression and DCN facilitation were not significantly different from control simulations (Figure 6C). CR acquisition was intact (control: 56; impaired: 55, McNemar’s *p* = 0.66). No significant differences were found in CR onset latencies (control: 139 ± 57 ms; impaired: 156 ± 57 ms, *p* = 0.23). PC and DCN intrinsic firing properties were similar between control and pathological simulations (Supplementary Figure 6).

Even imposing a more severe impairment on PC synaptic densities (25% reduction in *pf*-PC, 71% reduction in MLI-PC and 57% increase in IO-PC), baseline PC and DCN firing properties were unaltered (Supplementary Figure 6), and behavioral results were similar to the control condition were produced (Figure 6D).

## Discussion

In this work we used advanced SNN models to investigate whether different types of cerebellar lesions could modify EBCC in dystonia. The main observation is that specific cerebellar cortical lesions associated with dystonia, e.g., reduced olivocerebellar input and aberrant PC firing, can compromise EBCC learning. Conversely, other lesions, e.g., a change in the excitatory/inhibitory balance on PCs, were ineffective. These results support the concept that, although originally associated with basal ganglia dysfunction, dystonia might involve the cerebellum and can be regarded as a network disorder (Neychev et al., 2011; Shakkottai et al., 2016) affecting multiple brain areas and their connections. The fact that cerebellar microcircuit alterations demonstrated a causal role in some, but not in all cases, is akin to clinical reports showing inconstant EBCC changes in dystonia (Sadnicka et al., 2022) and suggests heterogeneity in the microscopic underpinnings of the disease.

### Reduced Olivocerebellar Input

IO spikes are thought to convey motor error signals to the cerebellum and are also implicated in the control of motor response timing (Marr, 1969; Albus, 1971; Jacobson et al., 2008). Thus, a reduced olivocerebellar input to PCs probably leads to impaired motor learning and abnormal temporal processing of somatosensory inputs conveyed from cortical regions to the cerebellum (Latorre et al., 2020). Indeed, our cerebellar SNN model predicted a deficit in CR generation and a delayed CR timing in simulations with reduced IO-PC communication, and the deficit significantly linearly increased with damage severity.

### Aberrant Burst-Firing Pattern in PCs

An altered PC burst-firing is known as a potential mechanism impairing cerebellar motor control and learning. The cerebellum is involved in the control of agonist and antagonist muscles, supposedly correlated with increase and decrease in DCN firing. From a physiological point of view, dystonia is characterized by abnormal co-contraction of agonist and antagonist muscles (Berardelli et al., 1998; Shakkottai, 2014). Therefore, an irregular PC and DCN activity caused by inappropriate burst-firing patterns might alter the timing of muscle contraction. We argue that, if normal PC tonic firing is transformed into abnormal burst-firing, certain muscles could be incorrectly facilitated due to DCN disinhibition, resulting in the simultaneous activation of agonist and antagonist muscles, causing dystonic movements. In EBCC, this might (at least partially) be reflected by the inappropriate timing of eyelid closure. This was indeed the case in simulations with our SNN model when PCs generated intrinsic burst-firing. The motor output crossed the threshold before the CR window and lost its proper shape consisting of alternating rising and decay phases. In other simulations, PC burst-firing was exaggerated by increasing the IO input. Again, this made DCN unstable, preventing the generation of proper and timed motor responses. In general, enhanced and unstable DCN activity could hinder the separation of signals to agonist and antagonist muscle, causing co-contractions and preventing efficient movement control with the consequent emergence of dystonic movements (Shakkottai, 2014).

### Imbalanced PC Synaptic Densities

In our SNN simulations, a partial imbalance in PC afferents from MLIs, *pfs* and IOs did not markedly affect EBCC, predicting that neuronal changes reported in dystonic mice (Vanni et al., 2015) would not induce CR learning alterations. Indeed, baseline firing properties, neural modulation and EBCC motor output were similar between control and pathological simulations. We hypothesize that this type of low-level modification in the cerebellum is associated with some types of dystonia where no EBCC alterations have been observed (Sadnicka et al., 2015).

The unchanged EBCC outcomes may be explained by the fact that the increased E/I balance on PCs, induced by altering the number of excitatory and inhibitory synapses, was counterbalanced by a stronger *pf*-PC LTD due to a stronger teaching signal from IO. Therefore, even if more excitatory synapses were present with respect to the inhibitory ones, the potentiated excitatory effect was depressed along the learning process, producing a CR curve comparable to the control condition.

### Impacts, Limitations and Future Work

Computational modeling provided us with a unique opportunity to evaluate the causal relationship between various microcircuit lesions (mostly identified in the experimental animals), network dysfunction, and emerging dystonic symptoms.

Nonetheless, spatial aspects of cerebellar lesions could not be considered, since our SNN model did not reproduce any specific cerebellar area but rather demonstrated a pathophysiological principle. Further work will be needed to differentiate the cerebellar regions according to detailed brain atlases and to address the impact of regional alterations. Indeed, Bologna and Berardelli suggested that a dysfunction of the whole cerebellum may cause abnormal movements and postures in many body parts, thus resembling generalized dystonia (Bologna and Berardelli, 2017), while topographically localized alterations of the cerebellum could be related to one affected body area in focal dystonia. Supporting this hypothesis, Raike and colleagues showed that limited regional damage to the cerebellum in mice induced focal dystonia, while extensive cerebellar damage led to generalized dystonia (Raike et al., 2013). In the present work, the cerebellar SNN model was used to simulate EBCC. Thus, it could correspond to cerebellar areas involved in eye movement control, e.g., the vermis of lobules V-VI (Cheng et al., 2014; Diedrighsen and Bastian, 2014). Impaired cerebellar function in the regions related to eye movement control could be associated with blepharospasm, a type of focal dystonia affecting eyelid movements. Indeed, structural (Ramdhani et al., 2014) and functional (Hutchinson et al., 2000; Kerrison et al., 2003) imaging studies report cerebellar changes in patients with blepharospasm. Eyelid CR acquisition during EBCC would be expected to be heavily compromised due to involuntary eyelid spasms. This is predicted in our simulations with IO-induced PC burst-firing, which produced unmodulated output and no CR acquisition. Accordingly, Valls-Sole and Defazio argue that no reports of EBCC are available in patients with blepharospasm since an abnormal eyeblink would interfere with EBCC training (Valls-Sole and Defazio, 2016). Conversely, in the types of dystonia affecting other body parts, functionalities of the cerebellar areas involved in eyelid movement control might be unaffected, resulting in normal EBCC acquisition. For example, DYT1 dystonia is characterized by an onset in one limb with task-specific dystonia at first, which later becomes less task-specific and progresses to other areas, becoming multifocal or generalized (Ozelius and Lubarr, 2016); however, the spread of dystonic symptoms to craniocervical muscles is rare, which could explain why EBCC is unimpaired in DYT1 patients (Sadnicka et al., 2015). In summary, the region-function mapping could be tested by using full-scale cerebellar model, applying region-specific lesions, and investigating the involvement of different cerebellar lobules in the various forms of dystonia.

We argue that a better understanding of the cerebellar role in dystonic motor networks would be fundamental to devise effective treatment strategies. In some studies, cerebellar transcranial magnetic stimulation (TMS) has shown positive effects on dystonic symptoms (Koch et al., 2014; Bradnam et al., 2015). However, as Miterko and colleagues argue, it is not clear when the cerebellum should be considered as a treatment target in dystonia (Miterko et al., 2019). For instance, TMS over the cerebellum would be a less invasive alternative to, for example, the deep brain stimulation (DBS) of the globus pallidus (Wichmann and DeLong, 2011). Again, the focality of TMS and the design of the applied pattern may play a crucial role that remains to be addressed.

Regardless of whether cerebellar abnormalities are primary or secondary to the compensation of neurodegeneration processes, the different types of cerebellar impairment during associative learning might help to distinguish dystonia forms. More extensive experiments, combined with the simulations described here (Table 1), could allow to define a more robust link between EBCC and different types of dystonia. Thus, EBCC could be used as a clinical non-invasive marker, as shown for other pathologies. For example, it was shown that EBCC outcomes could differentiate between Alzheimer’s Disease and cerebrovascular dementia (Woodruff-Pak et al., 1996).

**TABLE 1:** Summary table of experimental models of dystonia and corresponding in silico models.

Experimental model			In silico model	

References	Animal model	Observed abnormalities	Applied lesions in the SNN model (“pathology model”)	Effects of lesions in pathological simulations compared to control
Ledoux and Lorden, 2002; White and Sillitoe, 2017	Genetically dystonic (*dt*) rats; Ptf1a*^Cre^*;Vglut2*^fx/fx^* mice.	Reduced olivocerebellar input	Reduced IO-PC weight and reduced IO-PC number of connections (by 25%, 50% or 75%).	PC baseline = DCN baseline = PC irregularity = DCN irregularity = PC suppression ↓ DCN facilitation ↓ %CR ↓ CR time before US =

Ledoux and Lorden, 2002	Genetically dystonic (*dt*) rats	PC burst-firing pattern	Direct injection of intermittent spike trains in all PCs, during the whole simulation: 20 ms spike trains with 20–30 ms pauses; Reduced PC intrinsic current for basal discharge.	PC baseline: ↑ DCN baseline: ↓ PC irregularity: ↑ DCN irregularity: ↑ PC suppression: = DCN facilitation: = %CR: ↓ CR time before US: ↓

Hisatsune et al., 2013	*Itpr1* knockout mice	PC burst-firing with increased IO activity	Direct injection of intermittent spike trains to all IOs, during the whole simulation: 40 ms spike trains with 40 ms pauses.	PC baseline: = DCN baseline: ↑ PC irregularity: ↑ DCN irregularity: ↑ PC suppression: - DCN facilitation: - %CR: ↓ CR time before US: -

Vanni et al., 2015	Heterozygous torsinA knockout mice (Tor1a + /–) and human ΔGAG mutant torsinA transgenic mice (hMT)	Reduced *pf* synaptic contacts on PC distal dendrites; Increased IO synaptic contacts on PCs; Reduced GABAergic input to PCs.	Removed 14%, 25% of *pf*-PC connections; Increased 32%, 57% of IO-PC connections; Removed 39%, 71% of MLI-PC connections.	PC baseline: = DCN baseline: = PC irregularity: = DCN irregularity: = PC suppression: = DCN facilitation: = %CR: = CR time before US: =

*Specifically, reference experimental studies along with the animal models and observed neural abnormalities are reported on the left; equivalent lesions applied to the SNN model and effects during simulations are listed on the right. Pathological simulation outcomes are indicated as modifications of parameters related to neural activity and behavior (e.g., firing rate irregularity and %CR) with respect to control simulations: increased (↑), decreased (↓), unchanged (=), not available (-).*

A limitation of this study is that plasticity was modeled only at *pf*-PC synapses. However, plasticity at *pf*-MLI, PC-DCN and *mf*-DCN connections is also implicated in EBCC learning (Ohyama et al., 2006; Boele et al., 2018). Thus, a multiple-plasticity network could provide a more comprehensive view into EBCC learning abnormalities due to cerebellar dysfunctions associated with dystonia. For example, reductions in MLI-PC connections as in Vanni et al. (2015) might result in more evident EBCC impairments than in our simulations if MLI synapses were plastic. A further test could be to model, during EBCC, both Z- and Z+ PCs, which have different intrinsic excitability and are likely to undergo either simple spike suppression or facilitation, respectively (de Zeeuw, 2021).

Moreover, the simplified point neurons used here could be replaced by multicompartmental morphology-based models (Masoli et al., 2015; Masoli and D’Angelo, 2017), in order to focus on PC subcellular structural and functional abnormalities (Llinás and Sugimori, 1980; Stuart and Häusser, 1994; Womack and Khodakhah, 2004; Ohtsuki et al., 2012), which are often quite subtle in dystonia. PCs showed shortened primary dendrites and decreased spines on the distal dendrites (Zhang et al., 2011). In addition, specific ionic channels can be responsible for dystonic dysfunctionalities. The irregular spiking of mutant Purkinje cells found in cell-attached recording may be due to an alteration of calcium channels, Ca^2+^-dependent K^+^ currents, or both (Liu et al., 2020), or to the alteration of Na^+^-related mechanisms (Fremont et al., 2015). Consideration of these molecular mechanisms would pave the way to explore PCs as a therapeutic target (Cook et al., 2021): manipulating PC firing and cerebellar output may show great promise for treating dystonia (Liu et al., 2020).

Furthermore, our model included the cerebellar circuit only. This allowed to isolate the cerebellar contribution in the disorder and understand the impact of specific lesions. However, to investigate the role of the cerebellum in dystonia as a motor network disorder (Neychev et al., 2011), more complex systems involving multiple brain nodes could be used, e.g., including the basal ganglia and the motor cortex. By inducing impairments in the cerebellum, such brain models could provide insight into whether and how dysfunctional cerebellum affects the integrity of the full motor network. In addition, cerebellar neuromodulation treatments (e.g., DBS or TMS), for instance, specifically targeting altered PC activity with DBS (Brown et al., 2020), might be modeled, allowing us to monitor signal propagation through the whole motor network. This could help to better understand how the cerebellum modulates the activity of the basal ganglia through their subcortical connections (Bostan et al., 2010; Chen et al., 2014), or vice versa, and how it could be directly related to dystonia.

## Data Availability Statement

The datasets presented in this study can be found in online repositories. The names of the repository/repositories and accession number(s) can be found in the article/Supplementary Material. The code for running simulations and analyzing results from this article are available at the following repository: https://github.com/dbbs-lab/dystonia_ebcc.

## Author Contributions

AG designed the system and performed the simulations, analyzed the simulated data, and wrote the manuscript. AM performed the simulations, prepared the figures, and contributed in writing. ED’A supported in interpreting the results and defined the physiological aspects. CC coordinated the work, supported the design of the system and of the simulations, and wrote the manuscript. All authors contributed to the article and approved the submitted version.

## Conflict of Interest

The authors declare that the research was conducted in the absence of any commercial or financial relationships that could be construed as a potential conflict of interest.

## Publisher’s Note

All claims expressed in this article are solely those of the authors and do not necessarily represent those of their affiliated organizations, or those of the publisher, the editors and the reviewers. Any product that may be evaluated in this article, or claim that may be made by its manufacturer, is not guaranteed or endorsed by the publisher.

## Supplementary Material

Supplementary Figure 1 SDFs in PC population **(A)** and DCN motor output **(B)**, averaging across cell SDFs, for each trial. Increasing darkness of the lines corresponds to subsequent trials (the lightest is the first trial, the darkest is the last trial). Each trial starts with the CS onset and last 1000 ms in total. At 250 ms the US is delivered (purple vertical line) and co-terminates with CS at 280 ms (gray vertical line). The US causes a burst-pause in PCs, which then return to baseline; consequently, a pause-burst response is triggered in the DCN motor output, before the return to baseline. The signals change in the time window before US throughout learning, thanks to synaptic plasticity. Note that first 50 ms are cut since artifacts of SDF analysis. Dashed horizontal line indicates the baseline activity (in the first 100 ms), solid horizontal cyan line (right panel) represents the CR threshold.

Supplementary Figure 2 SDFs of downstate **(A)** and upstate **(B)** individual PCs, undergoing a significant SDF change (suppression/facilitation), in the last trial with respect to initial state (first 100 ms from CS onset) in the control condition. The thicker lines represent the average across cells. The frequency values are normalized with respect to the initial state activity (horizontal dashed lines at 0 Hz).

Supplementary Figure 3 Baseline firing rate **(A)**, and irregularity measured through the CV_ISI_ **(B)** of PCs (left) and DCN (right) in the case of reduced olivocerebellar input for the three damage levels, compared to the control condition. Cyan horizontal line indicates the mean, red horizontal line indicates the median, box edges – 1st and 3rd quartile, whiskers – minimum and maximum accepted values, excluding outliers (indicated as circles).

Supplementary Figure 4 Raster plots showing spiking activity of each cell (y axis) within PC population in the first trial of simulations with aberrant PC burst-firing, intrinsic **(A)** and IO-induced **(B)**. Gray vertical lines represent the CS onset and end, purple line marks the US onset.

Supplementary Figure 5 Baseline firing rate **(A)** and irregularity measured through the CV_ISI_ **(B)** of PC (left) and DCN cells (right) in the case of intrinsic and IO-induced PC burst-firing, compared to the control condition. Cyan horizontal line indicates the mean, red horizontal line indicates the median, box edges – 1st and 3rd quartile, whiskers – minimum and maximum accepted values, excluding outliers (indicated as circles).

Supplementary Figure 6 Baseline firing rate **(A)** and irregularity measured through the CV_ISI_ **(B)** of PC (left) and DCN cells (right) in the case of imbalanced PC synaptic densities, for mild and severe damage levels, compared to the control condition. Cyan horizontal line indicates the mean, red horizontal line indicates the median, box edges – 1st and 3rd quartile, whiskers – minimum and maximum accepted values, excluding outliers (indicated as circles).
