# Supplementary figures and images for: Cerebellum Involvement in Dystonia During Associative Motor Learning: Insights From a Data-Driven Spiking Network Model
Source: Front Syst Neurosci. 2022 Jun 16;16:919761. doi: 10.3389/fnsys.2022.919761 (PMC9243665; doi:10.3389/fnsys.2022.919761)

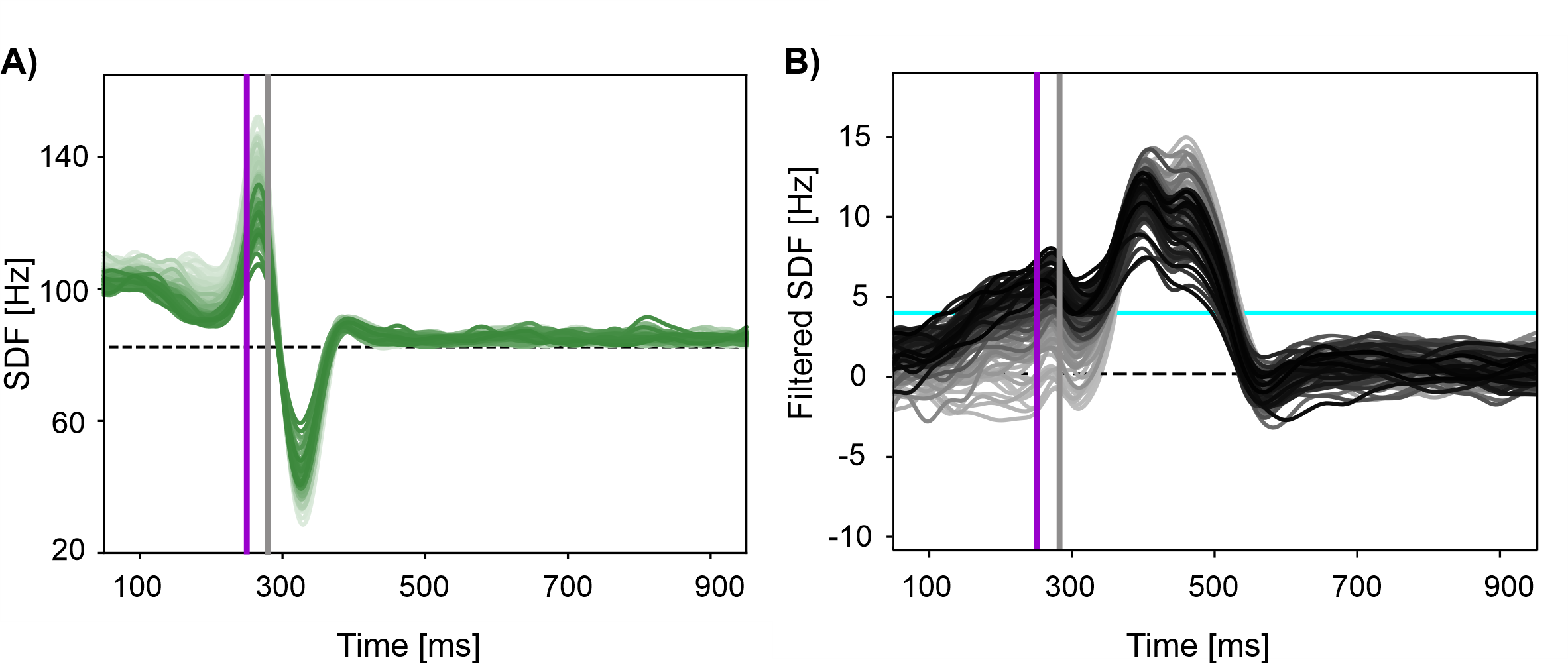

Supplement: Supplementary Figure 1 — SDFs in PC population (A) and DCN motor output (B), averaging across cell SDFs, for each trial. Increasing darkness of the lines corresponds to subsequent trials (the lightest is the first trial, the darkest is the last trial). Each trial starts with the CS onset and last 1000 ms in total. At 250 ms the US is delivered (purple vertical line) and co-terminates with CS at 280 ms (gray vertical line). The US causes a burst-pause in PCs, which then return to baseline; consequently, a pause-burst response is triggered in the DCN motor output, before the return to baseline. The signals change in the time window before US throughout learning, thanks to synaptic plasticity. Note that first 50 ms are cut since artifacts of SDF analysis. Dashed horizontal line indicates the baseline activity (in the first 100 ms), solid horizontal cyan line (right panel) represents the CR threshold. [file Image_1.TIF]

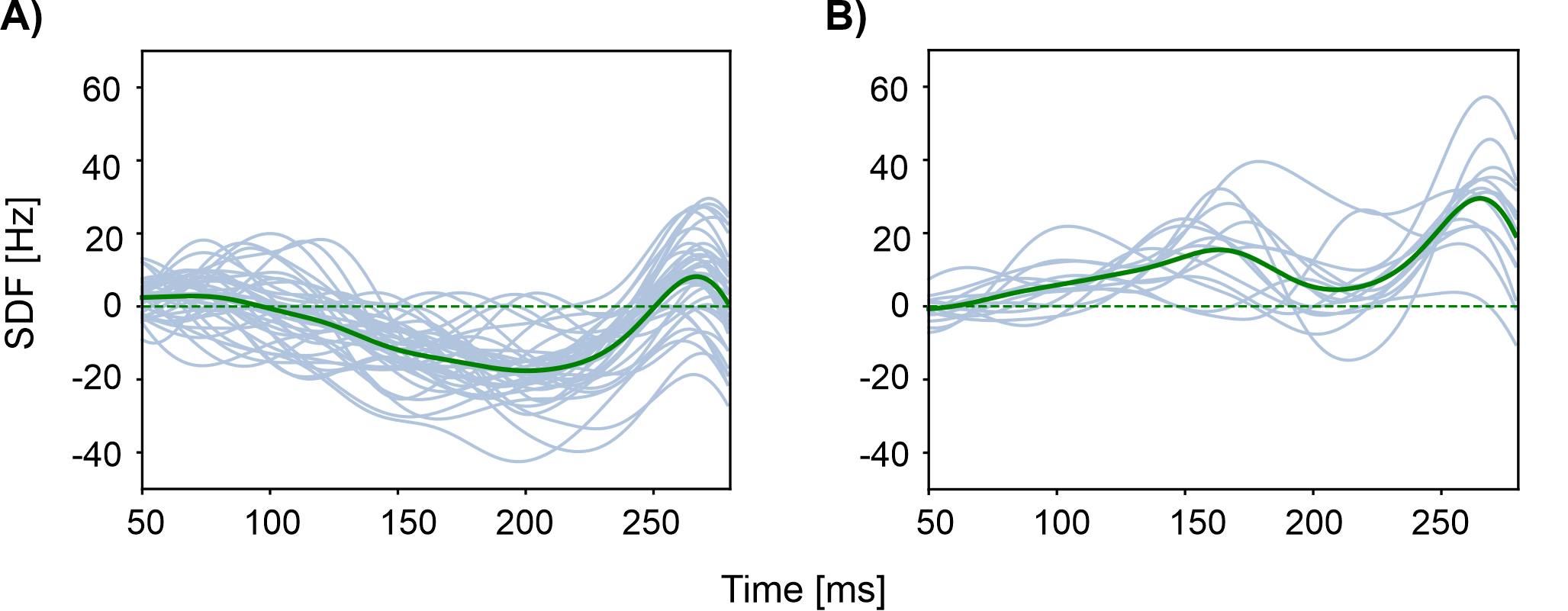

Supplement: Supplementary Figure 2 — SDFs of downstate (A) and upstate (B) individual PCs, undergoing a significant SDF change (suppression/facilitation), in the last trial with respect to initial state (first 100 ms from CS onset) in the control condition. The thicker lines represent the average across cells. The frequency values are normalized with respect to the initial state activity (horizontal dashed lines at 0 Hz). [file Image_2.TIF]

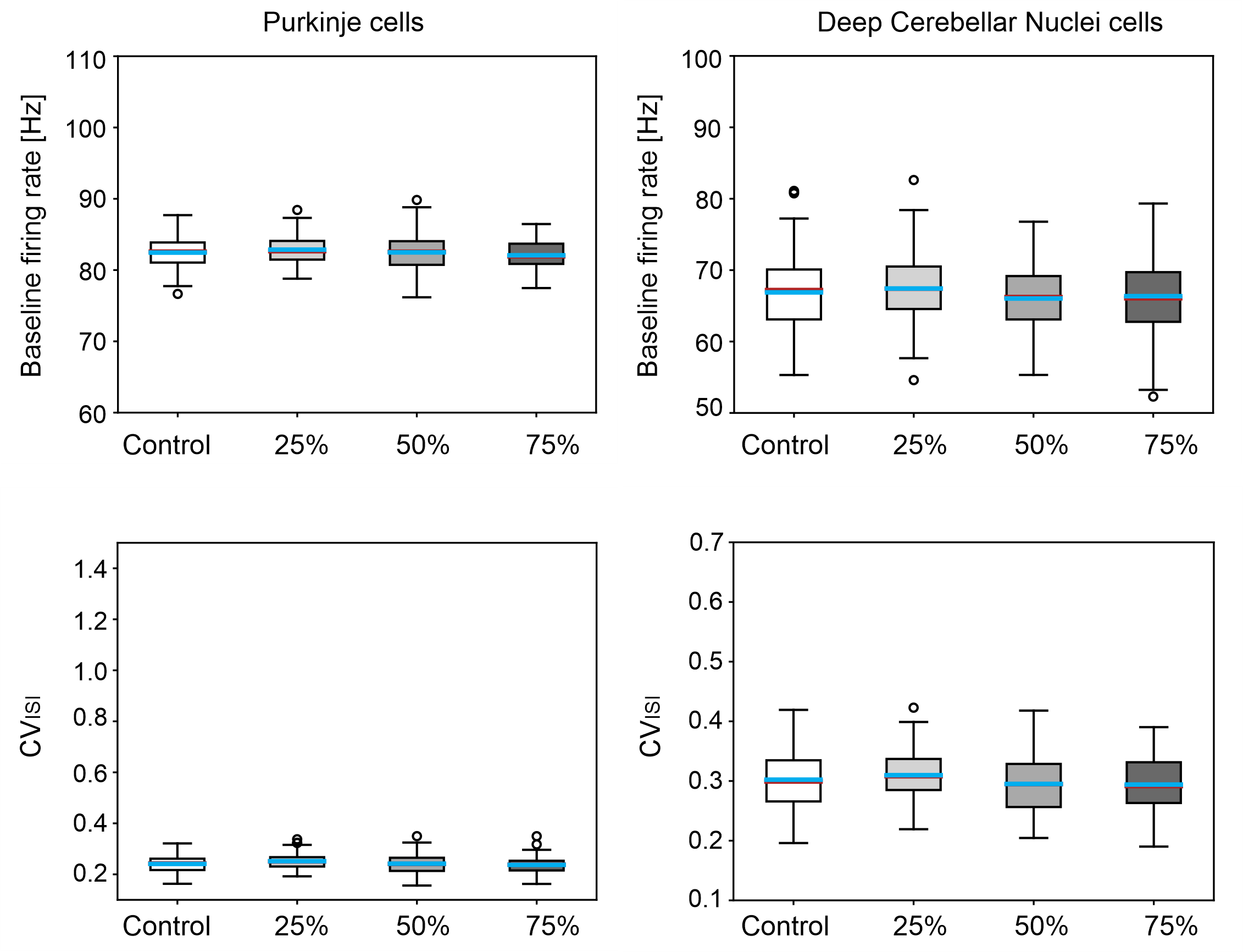

Supplement: Supplementary Figure 3 — Baseline firing rate (A), and irregularity measured through the CVISI (B) of PCs (left) and DCN (right) in the case of reduced olivocerebellar input for the three damage levels, compared to the control condition. Cyan horizontal line indicates the mean, red horizontal line indicates the median, box edges – 1st and 3rd quartile, whiskers – minimum and maximum accepted values, excluding outliers (indicated as circles). [file Image_3.TIF]

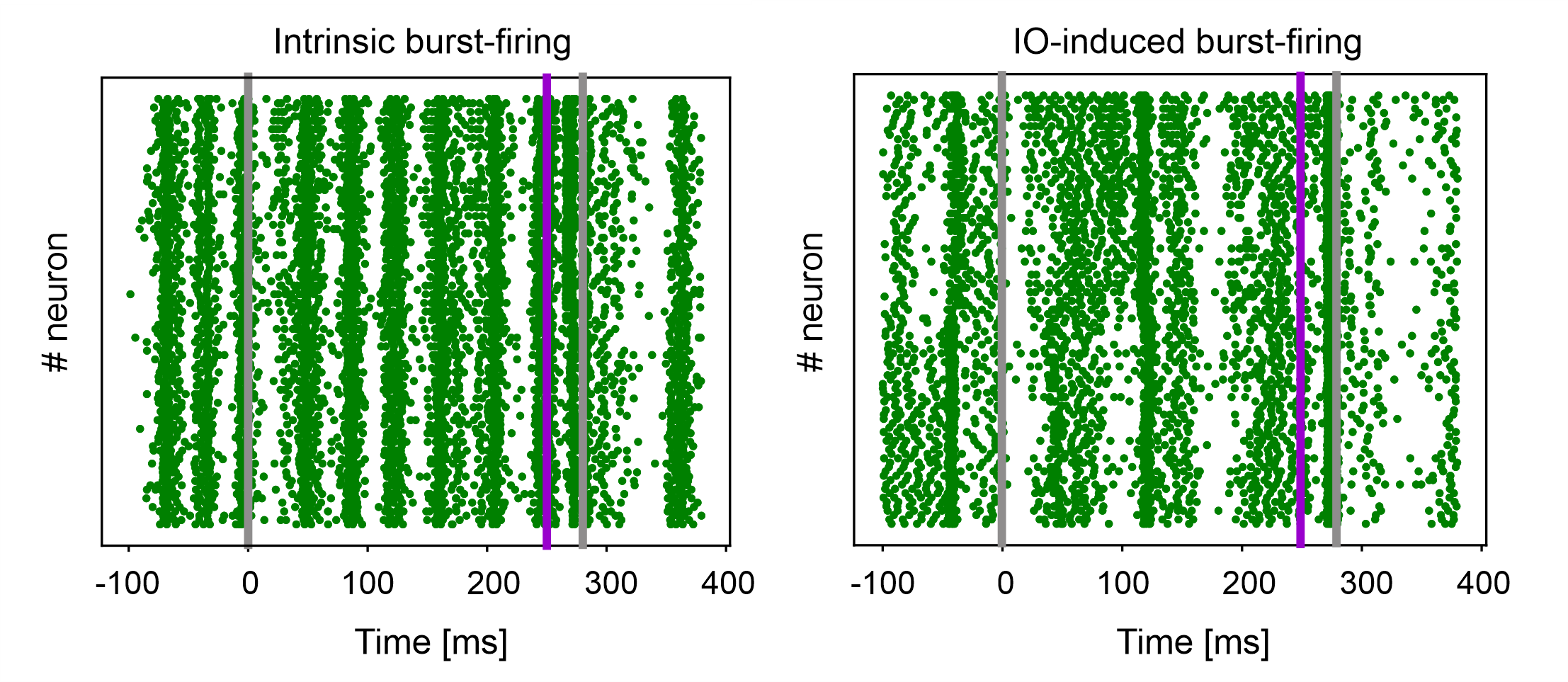

Supplement: Supplementary Figure 4 — Raster plots showing spiking activity of each cell (y axis) within PC population in the first trial of simulations with aberrant PC burst-firing, intrinsic (A) and IO-induced (B). Gray vertical lines represent the CS onset and end, purple line marks the US onset. [file Image_4.TIF]

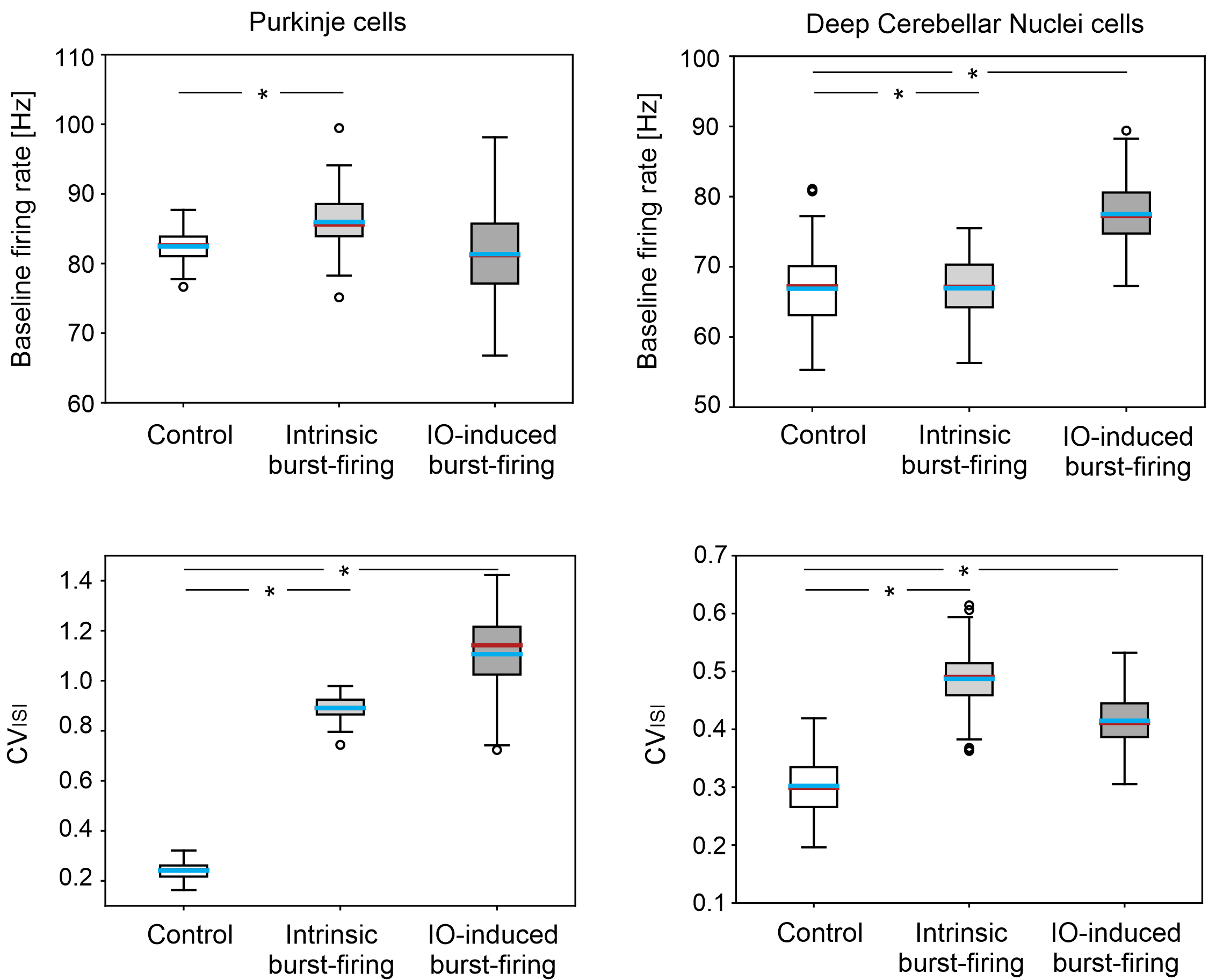

Supplement: Supplementary Figure 5 — Baseline firing rate (A) and irregularity measured through the CVISI (B) of PC (left) and DCN cells (right) in the case of intrinsic and IO-induced PC burst-firing, compared to the control condition. Cyan horizontal line indicates the mean, red horizontal line indicates the median, box edges – 1st and 3rd quartile, whiskers – minimum and maximum accepted values, excluding outliers (indicated as circles). [file Image_5.TIF]

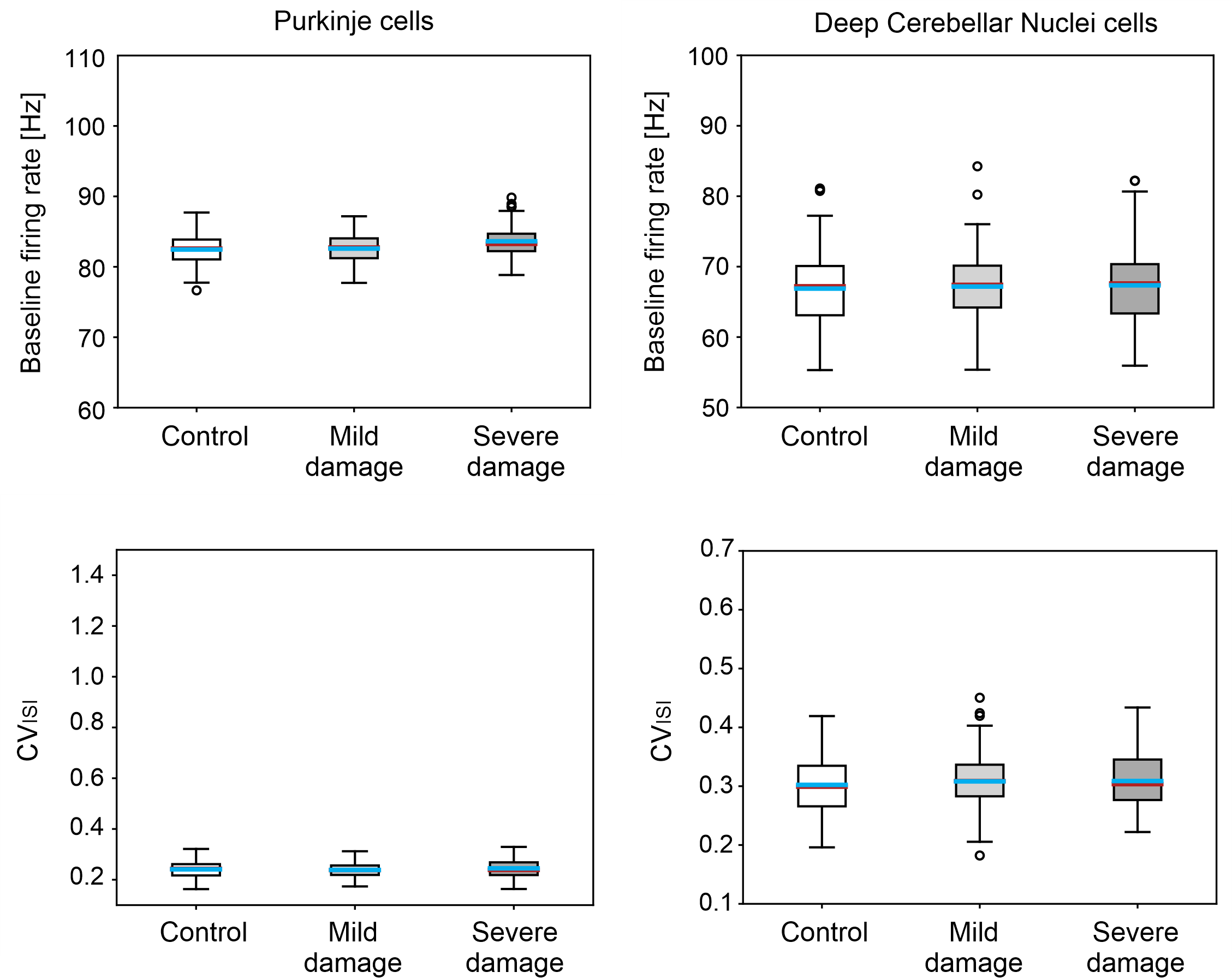

Supplement: Supplementary Figure 6 — Baseline firing rate (A) and irregularity measured through the CVISI (B) of PC (left) and DCN cells (right) in the case of imbalanced PC synaptic densities, for mild and severe damage levels, compared to the control condition. Cyan horizontal line indicates the mean, red horizontal line indicates the median, box edges – 1st and 3rd quartile, whiskers – minimum and maximum accepted values, excluding outliers (indicated as circles). [file Image_6.TIF]
